# Rehabilitation Strategies Following Posterolateral Corner Repair for Left Knee Dislocation With Multiligament Injury: A Case Report

**DOI:** 10.7759/cureus.56863

**Published:** 2024-03-25

**Authors:** Prajyot Ankar, Pratik Phansopkar

**Affiliations:** 1 Musculoskeletal Physiotherapy, Ravi Nair Physiotherapy College, Datta Meghe Institute of Higher Education & Research, Wardha, IND

**Keywords:** multiligament injury, posterolateral corner repair, case report, physiotherapy intervention, rehabilitation protocol, knee dislocation

## Abstract

This case report describes the rehabilitation of a 54-year-old female patient with a left knee dislocation and multiligament injury after surgery. The patient experienced persistent pain and difficulty with weight-bearing, leading to the need for surgical repair. The rehabilitation protocol included three phases: pain management, range-of-motion (ROM) restoration, muscle strength improvement, proprioception, and equilibrium promotion. Modalities like cryotherapy, compression, manual therapy, and a tailored exercise regimen were used. The patient's outcomes showed significant improvements post-rehabilitation, emphasizing the importance of structured physiotherapy interventions in recovery and functional restoration. The aim of the case report is to highlight the efficacy of a structured physiotherapy intervention protocol in facilitating recovery and functional restoration for patients with knee dislocation and multiligament injury post-surgery. Further research and evidence-based rehabilitation strategies are needed to improve outcomes in similar cases.

## Introduction

Posterolateral corner (PLC) injuries, resulting from varus impact on the flexed anteromedial knee, can lead to hyperextension and varus stress [1]. Athletic trauma, motor vehicle accidents, and falls are the primary causes of injuries to the PLC of the knee [2], despite being historically challenging to quantify due to diagnostic complexities [3]. However, advancements in magnetic resonance imaging (MRI) protocols and understanding of PLC anatomy have improved diagnostic accuracy [4]. According to a prospective MRI study conducted, PLC injuries were found in 16% of patients with ligamentous knee injuries [5]. PLC injuries rarely occur in isolation (5.7%) and are commonly associated with concurrent ligamentous injuries, particularly those involving the anterior cruciate ligament (ACL) or posterior cruciate ligament (PCL) [6]. The surge in motor vehicle accidents and sports-related injuries has played a significant role in the heightened prevalence of PLC injuries. These particular injuries often occur alongside ACL or PCL ruptures, while isolated PLC injuries are relatively rare. Despite their prevalence, PLC injuries can be easily overlooked or misdiagnosed. If left untreated, they may lead to chronic pain and residual instability. Therefore, it is crucial to detect and treat these injuries appropriately [6]. When PLC knee injuries are not sufficiently addressed, biomechanical overloading can lead to persistent pain, instability, and unfavorable surgical results in cruciate ligament reconstructions [7]. In the past, repairing the injured structures was typically reserved for acute cases. But because of the greater failure rates, which ranged from 40% to 6% in one cohort and from 37% to 9% in another, end-to-end isolated midsubstance repair is currently not advised [8]. The structures that sustain damage in knee hyperextension injuries are contingent upon the forces exerted during the injury and the individual's anatomical makeup. Generally, the initial strain or tear occurs in the posterior capsule, subsequently leading to injury in the PLC and ultimately affecting the PCL [9]. PLC injuries can occur due to various mechanisms, such as the application of a force directed toward the posterolateral aspect of the anteromedial tibia, excessive extension of the knee joint, or a combination of severe external rotation of the tibia while the knee is in a partially flexed position [10].

## Case presentation

Patient information

A 54-year-old woman presented with a chief complaint of persistent left knee pain and walking difficulties for the past 20 days. Approximately one month before seeking medical attention, she was in a state of general well-being until an incident occurred wherein she fell from a height of around 10 ft. Subsequent to this event, the patient experienced a sudden onset of nonprogressive, moderately intense, dull-aching pain localized to her left knee. This discomfort was exacerbated during movement and weight-bearing activities but demonstrated relief with rest and prescribed medications. Immediately following the incident, the patient found herself incapable of bearing weight on her left side, prompting a visit to a local healthcare facility. There, she received initial management comprising analgesics and the application of a long knee brace. Despite these measures, the persisting pain, coupled with ambulatory difficulties and a documented history of knee instability, led the patient to seek further evaluation and care at Acharya Vinoba Bhave Rural Hospital (AVBRH) on January 9, 2024. Following clinical assessment, a decision was made to proceed with surgical intervention, specifically a PLC repair on the left knee, conducted on January 11, 2024. Post-operatively, on the 12th day, the patient continued to experience ambulatory difficulties without external support. The nature of the pain persisted as a sudden onset, exacerbated during weight-bearing and movement, with relief observed upon rest and medication. The pain levels reported were 8/10 during activity and 5/10 while at rest, based on the Numerical Pain Rating Scale (NPRS). Due to the ongoing challenges in walking and persistent pain in the left knee, the patient has been referred for musculoskeletal physiotherapy as part of the comprehensive management plan.

Clinical findings

The patient's posture assessment revealed that standing with a walker was the highest attainable posture. The lateral view showed a forward-headed posture with a forward-flexed trunk and rounded shoulders. The patient's gait assessment showed that the patient walked with the aid of a walker and, notably, had no noticeable deformities. The patient relied on a long knee brace as an external appliance. Upon thorough inspection, no deformities in bony structure or alignment were noted, but quadriceps muscle wasting was evident. Palpation revealed tenderness, graded as Grade 1 (patient complains of pain). The end feel during knee joint movements was characterized by an empty end feel during knee flexion and a hard end feel during knee extension. Limb length measurements showed an apparent limb length of 90 cm on the right and 89 cm on the left, while the true limb length was 78 cm on the right and 77 cm on the left. Joint movement discrepancies were found between the right and left sides, with the right side demonstrating pain-free and complete movements. The left limb girth measurements above the knee from above the top of the knee cap were 36 cm at 3 in, 42 cm at 6 in, and 46 cm at 9 in. Below the knee from the superior apex of the patella, the corresponding measurements were 29 cm at 3 in, 32 cm at 6 in, and 25 cm at 9 in. The mobility assessment further revealed joint movement discrepancies between the right and left sides, with the right side consistently demonstrating pain-free and complete movements. In contrast, the left side exhibited patterns of painful and incomplete movements during knee flexion, knee extension, hip flexion, extension, abduction, adduction, internal rotation, and external rotation. The baseline range of motion (ROM) is shown in Table 1, and manual muscle testing is in Table 2.

**Table 1:** Baseline ROM ROM: Range of motion

Joint movement	Left active	Left passive	Right active	Right passive	Normal
HIP					
Flexion	0^0-^20^0^	0^0^-25^0^	0^0^-110^0^	0^0^-117^0^	0^0^-120^0^
Extension	0^0^-10^0^	0^0^-15^0^	0^0^-20^0^	0^0^-25^0^	0^0^-30^0^
Abduction	0^0^-15^0^	0^0^-20^0^	0^0^-35^0^	0^0^-40^0^	0^0^-45^0^
Adduction	0^0^-5^0^	0^0^-10^0^	0^0^-20^0^	0^0^-28^0^	0^0^-30^0^
Internal rotation	0^0^-10^0^	0^0^-15^0^	0^0^-37^0^	0^0^-40^0^	0^0^-45^0^
External rotation	0^0^-10^0^	0^0^-15^0^	0^0^-35^0^	0^0^-40^0^	0^0^-45^0^
KNEE					
Flexion	0^0^-20^0^	0^0^-25^0^	0^0^-120^0^	0^0^-125^0^	0^0^-135^0^
Extension	20^0^-0^0^	25^0^-0^0^	120-0^0^	125-0^0^	135-0^0^

**Table 2:** Baseline MMT MMT: Manual muscle testing; NA: not assessable Grade 5: Normal; Grade 4: movement against gravity and resistance; Grade 3: movement against gravity over (almost) the full range; Grade 2: movement of the limb but not against gravity; Grade 1: visible contraction without movement of the limb; Grade 0: no visible contraction

Muscle group	Muscle	Left side	Right side
Hip	Iliacus	2/5	5/5
Hip	Psoas major	2/5	5/5
Hip	Gluteus maximus	2/5	5/5
Hip	Gluteus medius	2/5	5/5
Hip	Gluteus minimus	2/5	5/5
Hip	Adductor longus	2/5	5/5
Hip	Adductor magnus	2/5	5/5
Hip	Adductor brevis	2/5	5/5
Hip	Pectineus	2/5	5/5
Hip	Tensor fasciae latae	3/5	5/5
Hip	Piriformis	3/5	5/5
Hip	Quadratus femoris	3/5	5/5
Hip	Obturator internus	3/5	5/5
Hip	Obturator externus	3/5	5/5
Hip	Gemellus superior	3/5	5/5
Hip	Gemellus inferior	3/5	5/5
Hip	Obturator externus	3/5	5/5
Hip	Obturator internus	3/5	5/5
Hip	Sartorius	3/5	5/5
Knee	Semitendinosus	NA	5/5
Knee	Semimembranosus	NA	5/5
Knee	Biceps femoris	NA	5/5
Knee	Rectus femoris	NA	5/5
Knee	Vastus lateralis	NA	5/5
Knee	Vastus medialis	NA	5/5
Knee	Vastus intermedius	NA	5/5

Investigations

**Figure 1:**
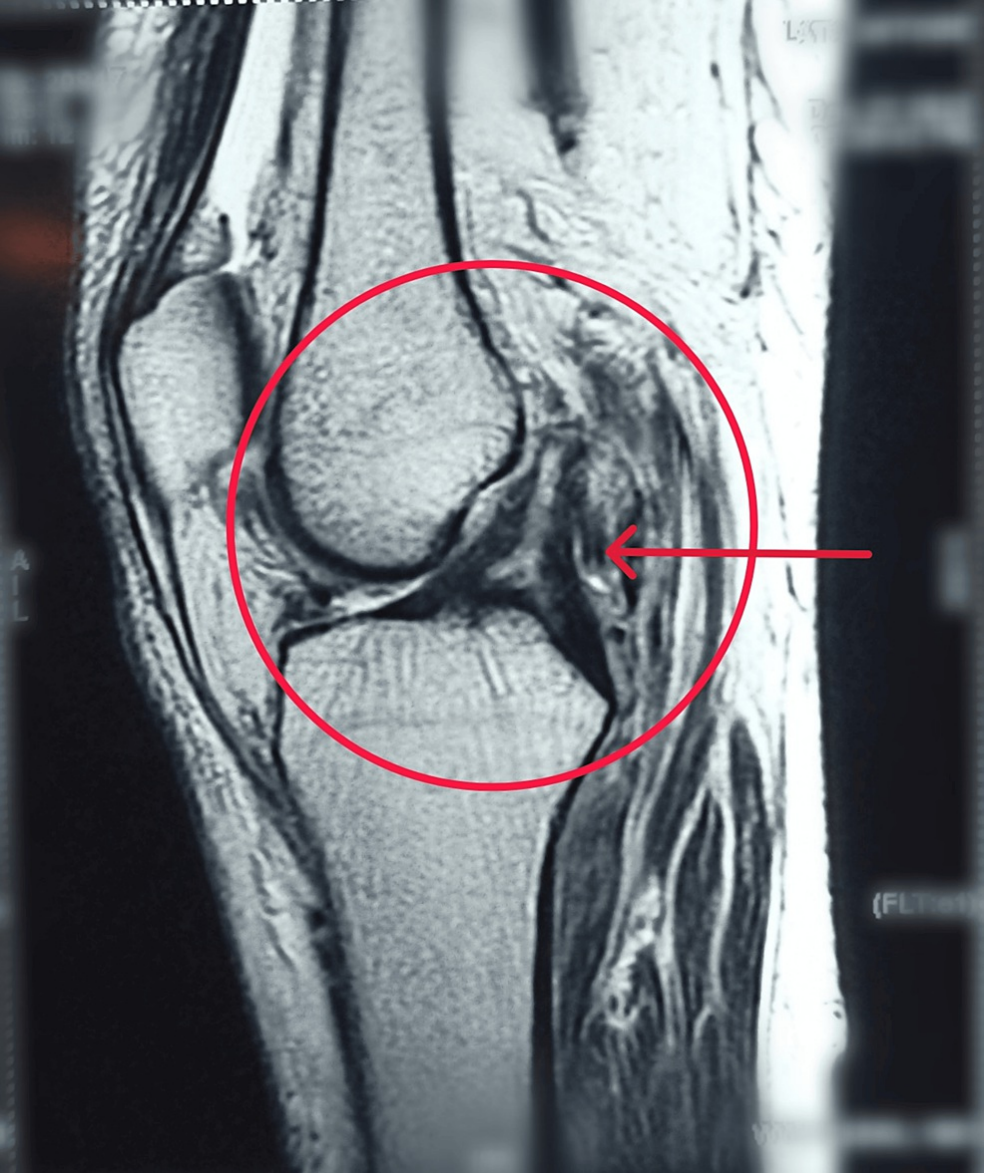
MRI showing ACL and PCL injury MRI: Magnetic resonance imaging; ACL: anterior cruciate ligament; PCL: posterior cruciate ligament

**Figure 2:**
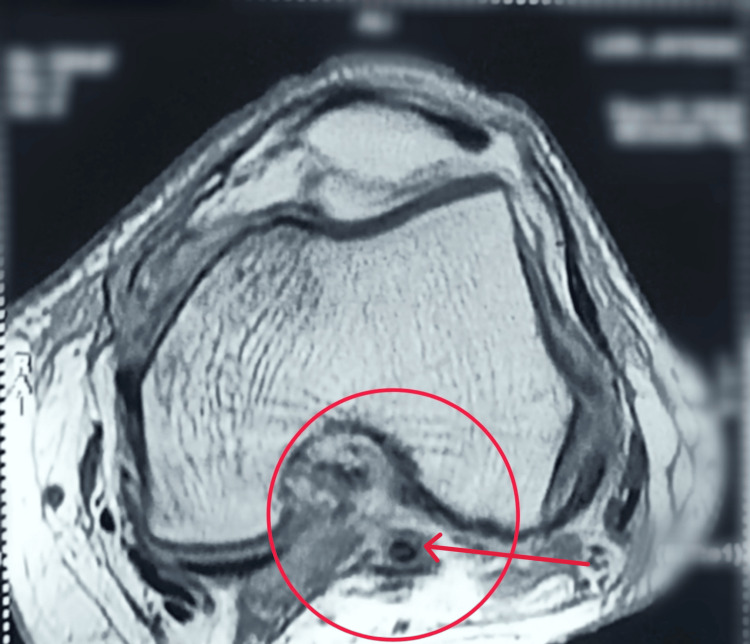
MRI showing ACL tear MRI: Magnetic resonance imaging; ACL: anterior cruciate ligament

The area under the red circle shows sagittal proton-density fast-spin-echo (PDFSE) with fat sat: There is bone marrow contusion of the posterolateral tibial plateau, and there is also fluid posterior to the popliteus, indicative of PLC injury (Figures 1, 2).

Physiotherapy intervention

**Figure 3:**
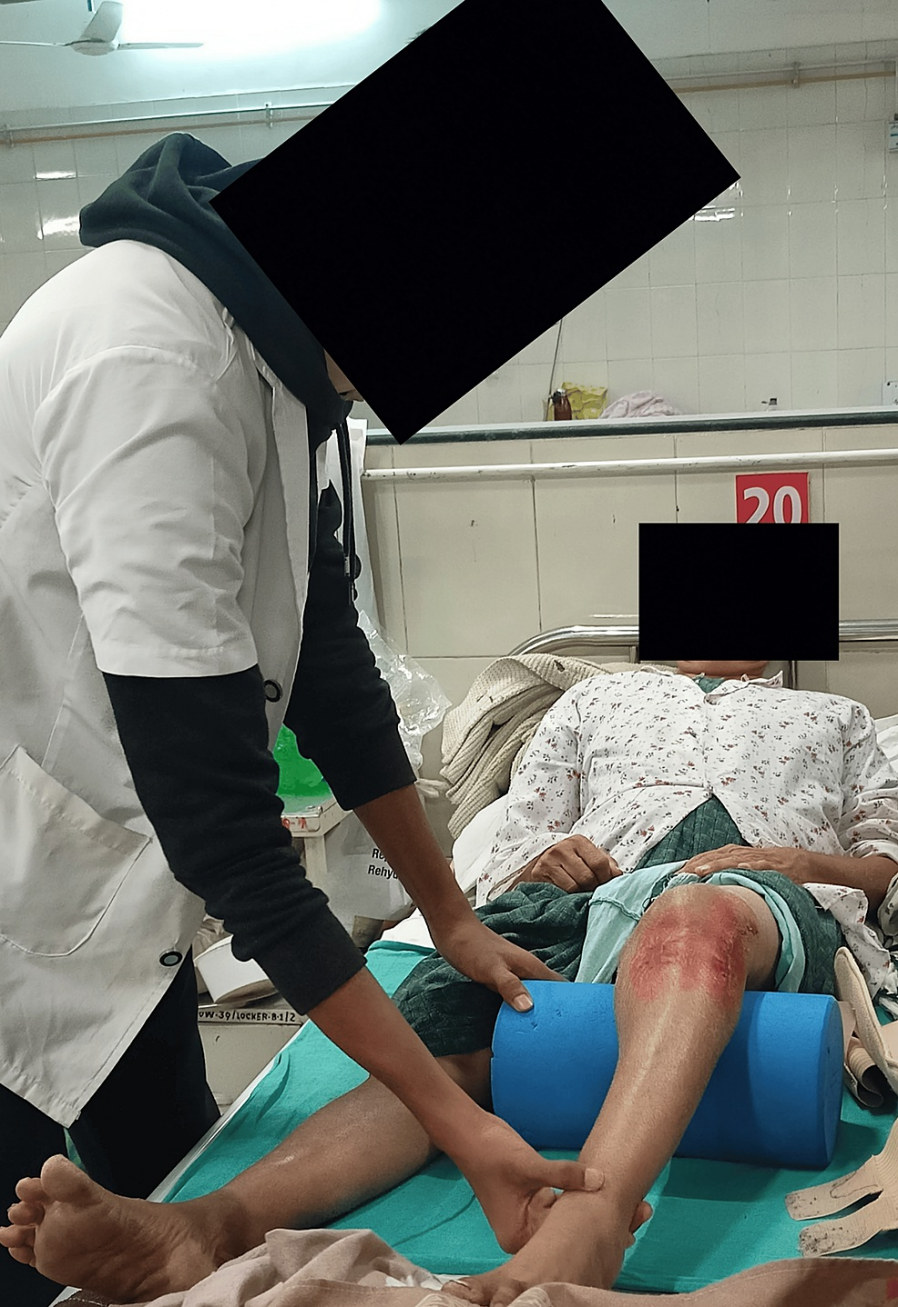
VMO strengthening exercise VMO: Vastus medialis oblique

**Figure 4:**
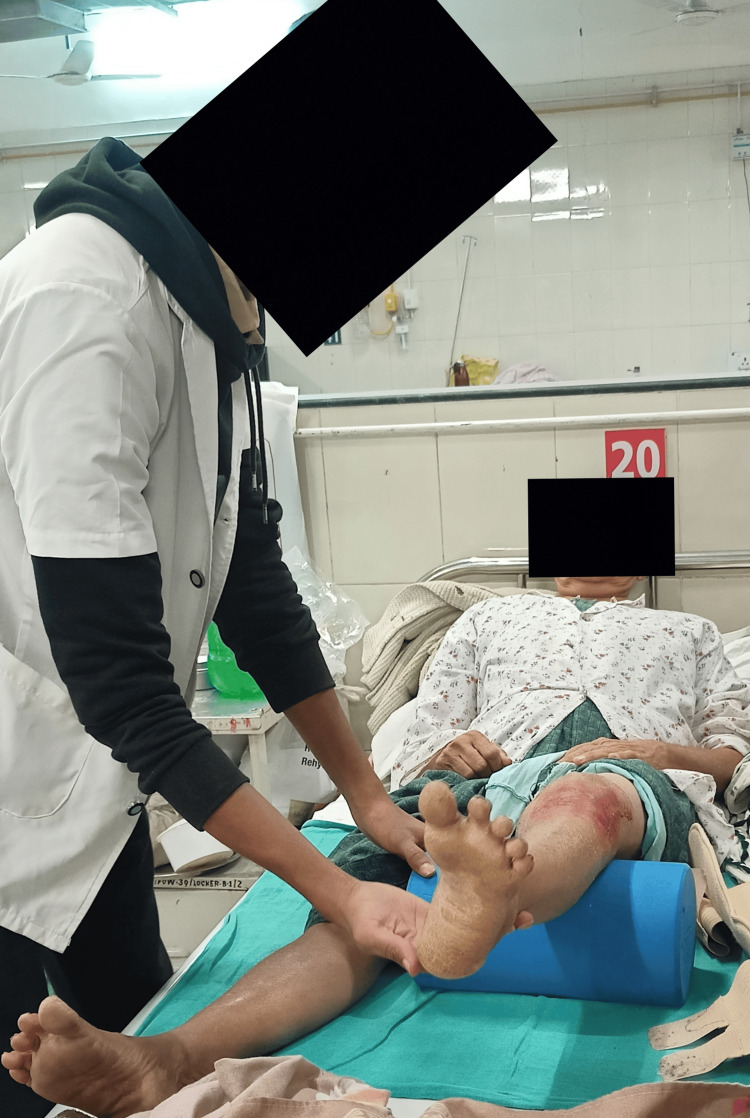
VMO strengthening exercise VMO: Vastus medialis oblique

Table 3 shows phase-wise rehabilitation [11]. Figure 3 and Figure 4 show the vastus medialis oblique strengthening exercise.

**Table 3:** Phase-wise physiotherapy rehabilitation

Phase of rehabilitation	Time point	Goals	Interventions	Precautions
Phase 1: Initial post-operative phase	0-6 weeks	Edema management, gentle range of motion, and activation of the quadriceps muscle	Cryotherapy, compression, and elevation	Avoid excessive hyperextension; avoid hamstring exercises with knee flexion; avoid tibial external rotation; and exercise caution with weight-bearing activities
			Repetitive ankle dorsiflexion and plantar flexion movements	
			Passive knee flexion or active-assisted knee flexion and extension	
			Patellofemoral mobilizations	
			Isometric quadriceps exercises and hamstring contractions exercises (30 repetitions, 5–6 times/day)	
			Performing straight leg raises (SLRs) while wearing a knee immobilizer	
			Knee immobilizer wear, except for exercises and quadriceps isometrics	
			By the end of the second week, the range of motion should be between 0° and 90°, while a full range of motion is expected to be achieved by the sixth week. 0° to 90° flexion by 2 weeks, full range of motion by 6 weeks	
			After 2 weeks, start vastus medialis oblique (VMO) strengthening (10 repetitions ×1 sets)	
Phase 2: Early weight-bearing Phase	6-12 weeks	Progressive weight-bearing, gait normalization, and continued quadriceps activation	A supine straight leg raise is performed without any lag in knee extension	A supine straight leg raise is performed without any lag in knee extension. Monitor for signs of effusion. Ensure gradual weight-bearing progression and normalize gait pattern quadriceps activation during gait
			The knee is positioned at 0° of extension and flexed beyond 120°	
			This exercise is followed by weight-bearing progression and gradual reduction in the use of crutches	
			Weight-shifting exercises with upper extremity support	
			Balance training	
			Gradual crutch weaning over 2 weeks	
			Quadriceps activation during loading phase of gait	
			Vastus medialis oblique (VMO) strengthening (10 repetitions ×3 sets )	
			Dynamic quadriceps (10 repetitions ×3 sets)	
			At the end of 11 weeks, gradually introduce open-chain exercises like hamstring curls, leg extensions, and calf raises	
Phase 3: Advanced rehabilitation	3-4 months	Progressive strength and functional exercises	Squat rack with 50% body weight (depth >70° knee flexion)	Ensure pain-free ambulation for jogging/running clearance, proper mechanics and control for single-limb squatting, gradual increase in exercise intensity and volume, control varus stresses during exercises
			Daily walking program (20 min, increase 5 min/week)	
			Initiation of jogging/running and plyometric exercises if cleared by surgeon	
			Stationary cycling with increased resistance (20 min, 3-5 times per week)	
			Lateral, forward and retro step-ups (gradually increasing height and repetitions)	
			Lunge progression (depth >70° knee flexion, control varus stresses)	
			Dynamic quadriceps with weight cuffs or quadriceps chair (12 repetitions ×3 sets)	
			Emphasis on eccentric strengthening to enhance muscle control and absorption of forces during deceleration	
			Progressive resistance training with higher loads	

Outcome measures

Lower extremity functional scale (LEFS): The pre-rehabilitation score was 15/80, and the post-rehabilitation score was 53/80. The knee injury and osteoarthritis outcome score (KOOS) is shown in Table 4. Post-rehabilitation range of motion is in Table 5. Post-rehabilitation manual muscle testing is in Table 6.

**Table 4:** Knee injury and osteoarthritis outcome score (KOOS) KOOS: Knee injury and osteoarthritis outcome score; ADL: activities of daily living; Sports/Rec: sport and recreation function; QOL: quality of life

KOOS scale	Pre-rehabilitation score	Post-rehabilitation score
KOOS Symptoms	18	71
KOOS Pain	11	64
KOOS ADL	7	68
KOOS sports/rec	0	35
KOOS QOL	38	50

**Table 5:** Post-rehabilitation ROM ROM: Range of motions of the joints

Joint movement	Left active	Left passive
HIP		
Flexion	0^0-^70^0^	0^0^-90^0^
Extension	0^0^-25^0^	0^0^-40^0^
Abduction	0^0^-35^0^	0^0^-40^0^
Adduction	0^0^-20^0^	0^0^-26^0^
Internal rotation	0^0^-35^0^	0^0^-40^0^
External rotation	0^0^-38^0^	0^0^-45^0^
KNEE		
Flexion	0^0^-90^0^	0^0^-95^0^
Extension	90^0^-0^0^	95^0^-0^0^

**Table 6:** Post-rehabilitation manual muscle testing Grade 5: Normal; Grade 4: movement against gravity and resistance; Grade 3: movement against gravity over (almost) the full range; Grade 2: movement of the limb but not against gravity; Grade 1: visible contraction without movement of the limb; Grade 0: no visible contraction

Muscle group	Muscle	Left side
Hip	Iliacus	4/5
Hip	Psoas major	4/5
Hip	Gluteus maximus	4/5
Hip	Gluteus medius	4/5
Hip	Gluteus minimus	4/5
Hip	Adductor longus	4/5
Hip	Adductor magnus	4/5
Hip	Adductor brevis	4/5
Hip	Pectineus	4/5
Hip	Tensor fasciae latae	4/5
Hip	Piriformis	4/5
Hip	Quadratus femoris	4/5
Hip	Obturator internus	4/5
Hip	Obturator externus	4/5
Hip	Gemellus superior	4/5
Hip	Gemellus inferior	4/5
Hip	Obturator externus	4/5
Hip	Obturator internus	4/5
Hip	Sartorius	4/5
Knee	Semitendinosus	4/5
Knee	Semimembranosus	4/5
Knee	Biceps femoris	4/5
Knee	Rectus femoris	4/5
Knee	Vastus lateralis	4/5
Knee	Vastus medialis	4/5
Knee	Vastus intermedius	4/5

## Discussion

The physiotherapy interventions employed in this case have yielded positive outcomes, as evidenced by the post-rehabilitation outcome measures. The treatment plan, consisting of three distinct phases tailored to the patient's needs at various stages of recovery, has demonstrated effectiveness in addressing the challenges presented post-knee surgery. The rehabilitation program, evaluated through the LEFS and the KOOS, demonstrated notable enhancements in functional limitations, pain, daily tasks, physical activities, and overall well-being following the rehabilitation process. These positive outcomes demonstrate the program's effectiveness in restoring function and reducing pain. Early intervention following knee surgery has been underscored as crucial for optimizing recovery time and functional outcomes. Larsen et al. investigated the effects of early post-operative intervention in knee surgery, aligning with our approach of initiating rehabilitation during the initial post-operative phase [12]. The emphasis on edema management, gentle ROM, and activation of the quadriceps muscle during this phase corresponds to the findings emphasizing the significance of early rehabilitation in improving recovery time and functional outcomes [12].

Furthermore, the role of early rehabilitation in promoting optimal recovery and functional outcomes is supported by the systematic review conducted by Zheng et al. [13]. Their findings reinforce our phased approach to rehabilitation, highlighting the importance of early intervention in facilitating recovery and promoting independence in ambulation [13]. Pain management strategies, including multimodal approaches such as cryotherapy, compression, and manual therapy, have played a pivotal role in reducing pain intensity and improving functional recovery post-surgery. Studies by Lewis et al. [14], Quesnot et al. [15], and Chunduri and Aggarwal [16] have demonstrated the effectiveness of multimodal pain management strategies in knee surgery. These studies have shown that the combination of cryotherapy, compression, and manual therapy not only reduces pain but also facilitates an earlier return to functional activities and improves patient satisfaction. Our utilization of these modalities and manual therapy techniques is further supported by the findings of these studies, which highlight their efficacy in alleviating pain and promoting engagement in therapeutic exercises and activities. Moreover, manual therapy techniques have been shown to improve joint mobility and reduce pain post-surgery, complementing our pain management strategies. Farr et al. focus on manual therapy techniques for post-operative knee pain, supporting our approach to incorporating manual therapy into the rehabilitation program to enhance joint mobility and alleviate pain [17]. The inclusion of balance training and proprioceptive exercises in the rehabilitation program has also contributed to positive outcomes post-knee surgery. LaPrade et al. demonstrated the benefits of balance training in enhancing joint stability and functional recovery, which aligns with our emphasis on balance training during the early weight-bearing phase [18]. Additionally, proprioceptive exercises have been shown to improve joint awareness and stability, supporting their inclusion in the rehabilitation program to enhance functional outcomes post-knee surgery, as emphasized by Domínguez-Navarro et al. [19].

## Conclusions

The individual suffering from a left knee dislocation and multiligament injury received rehabilitation through physiotherapy techniques. This regimen resulted in enhancements in ROM, muscle strength, and overall functional ability. The program used modalities like cryotherapy, compression, and manual therapy, along with exercises targeting pain, edema control, ROM, muscle activation, balance, and proprioception. The findings are consistent with prior research, underscoring the significance of organized physical therapy in enhancing recuperation and facilitating functional improvement following knee surgery. Further research and evidence-based rehabilitation strategies are needed to enhance patient outcomes in similar cases.
